# Education for appropriate seatbelt use required for early-phase pregnant women drivers

**DOI:** 10.1038/s41598-020-74730-5

**Published:** 2020-10-19

**Authors:** Kyoko Hanahara, Masahito Hitosugi, Yumiko Tateoka

**Affiliations:** 1 Department of Clinical Nursing, Maternal Nursing and Midwifery, Shiga University of Medical Science, Tsukinowa, Seta, Otsu, Shiga 520-2192 Japan; 2 Department of Legal Medicine, Shiga University of Medical Science, Tsukinowa, Seta, Otsu, Shiga 520-2192 Japan

**Keywords:** Health care, Medical research

## Abstract

Considerable numbers of pregnant women do not understand the correct way to use seatbelts; thus, they are inappropriately restrained when wearing seatbelts. To improve appropriate seatbelt wearing by pregnant women vehicle passengers, we examined their use by pregnant women drivers and the independent factors influencing appropriate use. We undertook a cross-sectional survey of 1,000 pregnant women in Shiga Prefecture, Japan. Among 774 returned questionnaires, we analysed those of 680 pregnant women who always wore a seatbelt. The mean participant age was 31.4 ± 5.0 years and mean gestational age 26.2 ± 8.2 weeks; 97.7% of subjects always wore a seatbelt; 86.9% wore a seatbelt correctly and 13.1% incorrectly. Multivariate analysis indicated that receiving information about correct seatbelt use (odds ratio, 2.25; *P* < 0.005) and gestational age (odds ratio, 1.06; *P* < 0.001) were significant independent factors for correct seatbelt use. Providing information about correct seatbelt use during the early term is required for pregnant women to protect both the mother and fetus.

## Introduction

Maternal health has recently been accorded higher priority: the World Health Organization recommends promoting and protecting maternal and perinatal health^1^. Therefore, in addition to general health promotion, safety promotions to avoid injuries during pregnancy should be supported. Trauma during pregnancy is a leading cause of maternal and fetal morbidity and mortality. According to one retrospective study of females of childbearing age, pregnancy did not induce improved safety behaviour^2^. Another retrospective cohort study using a trauma database suggested that pregnant women were almost twice as likely to die after trauma^3^. Post-traumatic fetal mortality has been estimated at around 3–7 deaths per 100,000 live births^4^. One systematic review of the literature from 2006 to 2016 revealed that motor vehicle collisions (MVCs) were the most frequent cause of blunt trauma^5^. In the United States, approximately 130,000 women annually were reportedly involved in MVCs during the second half of their pregnancy^6^. Of the survivors, according to that study, 300–3,800 sustained fetal loss. According to Connolly et al., 6%–7% of pregnant women underwent a subtype of traumatic injury during pregnancy; approximately two-thirds of such injuries occurred as a result of traffic accidents^7^. Owing to the lack of nationwide statistics regarding pregnant women involved in traffic accidents in Japan, the actual numbers in this area are unknown. A recent questionnaire survey revealed that 2.9% of pregnant women were involved in vehicle collisions in Japan^8^. Thus, avoiding traffic injuries during pregnancy should receive greater priority for the health of both the mother and fetus.

For vehicle occupants, wearing three-point seatbelts reduces the mortality and extent or severity of injuries. Wearing seatbelts by drivers and vehicle passengers is therefore legally required in many countries. According to some studies, wearing a seatbelt during pregnancy has reportedly better fetal outcomes after MVCs than not wearing one^9,10^.

However, in addition to a lack of scientific evidence from medical and engineering perspectives, there has, been the widespread misunderstanding that wearing a seatbelt could be harmful for the abdomen. Thus, a substantial number of pregnant women do not use a seatbelt in a vehicle. Accordingly, a series of front and rear impact sled tests using anthropometric models of pregnant women were performed to study the mechanisms of injuries in pregnant drivers^11,12^. With frontal impacts, the unbelted dummy’s abdomen directly hit the steering wheel; with rear impacts, a similar phenomenon was evident following a rebound. Subsequently, higher values of abdominal pressure (indicating intra-uterine pressure) were obtained with a pregnant woman dummy even with low-velocity impacts^11,12^. However, wearing a three-point seatbelt reduced abdominal pressure or prevented contact with the steering wheel during frontal and rear collisions. Thus, the positive effects of wearing a seatbelt were biomechanically confirmed.

To increase seatbelt use among vehicle passengers, the American College of Obstetricians and Gynecologists recommends wearing a seatbelt correctly toward reducing the risk of injury to both the mother and fetus^13^. In 2008, Japan’s National Police Association also recommended that pregnant women travelling in vehicles should wear a seatbelt. As result, approximately 90% of pregnant women and 94.8% of drivers in Japan in 2009 always wore a seatbelt; 89.2% of front-seat passengers did so in 2013^8,14^. Health-care providers have undertaken related interventions. One investigation determined that half of pregnant women had received counselling regarding seatbelt use from their prenatal care providers^15^. Another report observed that 57.8% of pregnant women had received information about seatbelt use from a doctor or nurse in a rural clinic^16^. Some studies have determined that pregnant women who received information concerning seatbelt use were significantly more likely to understand proper placement and wear seatbelts correctly^17–19^. Other investigations have found that positive beliefs about seatbelts increased the likelihood of wearing them^20,21^.

Education has therefore contributed to seatbelt use by pregnant women passengers. However, around one in four pregnant women did not understand the correct method for using a seatbelt; thus, pregnant women reported that they felt inappropriately restrained when using a seatbelt^14^. Therefore, for the safety of pregnant women, in addition to wearing a seatbelt, enhancing appropriate use is important. To promote correct seatbelt use by pregnant women, issues that positively affect such use need to be considered as part of the education. No reports, however, have examined the factors that influence appropriate seatbelt use by pregnant women vehicle passengers.

The present study aimed to clarify the situation regarding seatbelt use by pregnant women drivers in Japan. We also aimed to identify independent factors that influence appropriate seatbelt use. Our results should be useful for interventions to improve appropriate seatbelt wearing by pregnant women vehicle passengers.

## Methods

### Study subjects

This study adopted a cross-sectional design. The study sites were six obstetrics clinics with obstetrics facilities in the cities of Otsu, Kusatsu, and Konan in south-west Shiga Prefecture. The prefecture is located in central Japan and includes the country’s largest lake (Biwa), which covers about one-sixth of the prefecture. In 2018, Shiga’s population was 1,412,881 (including 11,598 newborns). South-west Shiga had a population of 433,668 (6,972 newborns). Many pregnant women in south-west Shiga normally use private motor vehicles when travelling to a clinic or going shopping or commuting. This study targeted pregnant women who attended maternal health check-ups or mothers’ classes. Japan’s Maternal and Child Health Law stipulates that pregnant women have to receive health education through such classes. From August to December 2018, we distributed a questionnaire to 1,000 pregnant women. We excluded subjects who were unable to read or write Japanese.

### Survey

After requesting and obtaining permission from each obstetrics clinic and the hospital director, we distributed a self-administered questionnaire to pregnant women in the maternity facility reception area or mother’s classroom. After completion, questionnaires were placed in a box at the reception area in each facility. The questionnaire included an explanation of the research objectives. The questionnaire carried a statement assuring participants of the survey’s anonymity and that submission of the completed questionnaire constituted providing consent. Thus, informed consent was obtained from all subjects. All methods were carried out in accordance with relevant guidelines and regulations. The Research Ethics Committee of Shiga University of Medical Science approved this study (No. 29–245).

The questionnaire included items on the following topics:

1. General subject characteristics: age, body mass index (BMI);
2. Information about pregnancy: primipara or multipara, gestational age;
3. Favoured seat in the vehicle: driver’s, front passenger, or rear seat;
4. Driver or vehicle information: driving history, type of vehicle (normal sedan, small sedan [Kei car], sports utility vehicle [SUV], one-box, other), driving habit (almost every day, two to three times a week, one to two times a week, never);
5. Frequency of seatbelt use: always, sometimes, or never;
6. Understanding law regarding seatbelt use: no obligation to wear for any seats, required only for driver’s seat, required for front seats only, or required for all seats;
7. Information about correct seatbelt use during pregnancy: received or not received; if received, who provided the information.

**Figure 1:**
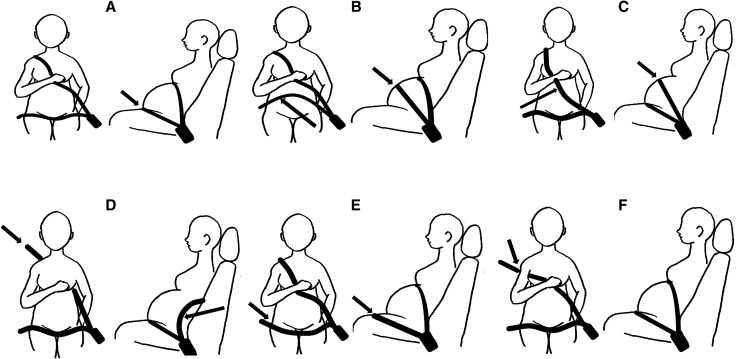
Various ways of wearing a seatbelt.

An examination was made of the current way of using a seatbelt. Illustrations of different ways of using a seatbelt appeared in the questionnaire, and each subject selected one that corresponded to her use (Fig. 1). The methods were classified as follows: lap belt crossing the lower abdomen, shoulder belt crossing between the breasts (A); lap belt crossing over the abdomen (B); shoulder belt crossing over the abdomen (C); wearing only the lap belt (D); lap belt crossing over the thighs (E); shoulder belt crossing under the armpit (F). With method A, the seatbelt was used appropriately, so subjects selecting this were regarded as correct seatbelt users.

### Statistical analysis

We categorized the subjects in terms of correct and incorrect seatbelt use. With those two groups, we compared the obtained variables. We used the χ^2^ test to compare categorical data. We employed the *t* test to compare continuous variables. To identify independent factors that influenced correct seatbelt use, we applied multivariate logistic regression analysis. We included all the factors that appear in Table 2 in the multivariable model. We conducted the analyses using SPSS, version 25 for Windows. We considered *P* values < 0.05 statistically significant.

## Results

### Basic subject details

In all, 774 questionnaires (77.4% of targeted subjects) were returned; 36 questionnaires were incomplete and therefore excluded. Accordingly, we analysed the data from 738 pregnant women. The mean age was 31.4 ± 5.0 years (range, 18–44 years). The average BMI was 20.6 ± 2.6. Among the participants, 408 (55.3%) were primipara (mean age, 30.1 ± 5.1 years); 330 (44.7%) were multipara (mean age, 33.0 ± 4.4 years). The mean gestational age was 26.2 ± 8.2 weeks: 52 (6.8%) subjects were less than 13 weeks; 318 (43.1%) were less than 28 weeks; 370 (50.1%) were 28 weeks or more.

### Driving habit and seatbelt use

**Figure 2:**
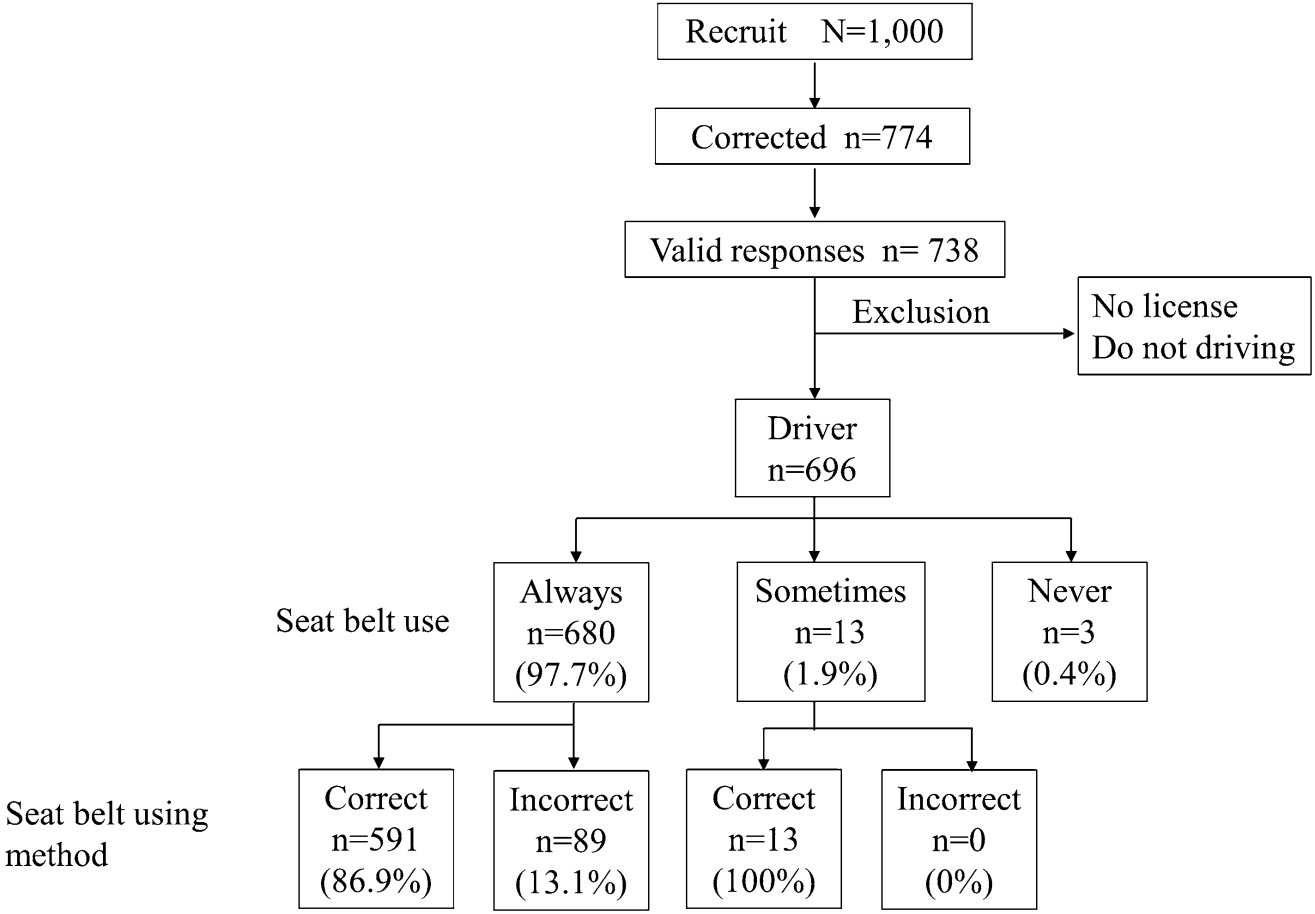
Overview of study subjects.

Most subjects (98.2%) had a driving license. The mean driving history was 11.4 ± 6.6 years. Almost half the participants (54.6%) drove a vehicle every day; 35.6% drove two to three times a week; 6.6% drove one to two times a month. We categorized 696 subjects as vehicle drivers (Fig. 2). Regarding the type of vehicle, almost half (51.5%) drove a Kei car; 27.0% drove a normal sedan type, 16.4% a one-box, 4.6% SUVs, and 0.5% other vehicles.

Among the drivers, 680 subjects (97.7%) always wore a seatbelt; 13 (1.9%) did so sometimes and three never did (0.4%). With the 680 drivers who always wore a seatbelt, 591 (86.9%) did so correctly; 89 (13.1%) did so incorrectly (Fig. 2). Among the incorrect users, the most common method was with the lap belt crossing over the abdomen (7.4%); that was followed by the lap belt crossing over the thighs (2.9%) and the shoulder belt crossing over the abdomen (1.1%; Table 1).

**Table 1:** Methods employed by drivers who always used a seatbelt (n = 680).

Variable	n (%)
Correct seatbelt use	591 (86.9%)
Lap belt crossing over the abdomen	51 (7.4%)
Shoulder belt crossing over the abdomen	7 (1.1%)
Wearing only the lap belt	3 (0.4%
Lap belt crossing over the thighs	20 (2.9%)
Shoulder belt crossing under the armpit	5 (0.9%)
Other	3 (0.4%)

### Awareness of seatbelt law and information sources

Among the 696 drivers, 68.2% fully understood that the law required seatbelt use for all seats; 2.2% believed that it was required only for drivers, 23.6% for drivers and front-seat passengers only, and 6.0% not necessary for any seats. Among the drivers, 35.5% indicated that they had received information about correct seatbelt use. Regarding the information source, only 6.8% of drivers had received the information from health-care professionals; among those, midwives were the source in 93.6%.

### Comparison of correct and incorrect seatbelt use

We compared the obtained variables among the 591 correct seatbelt users and 89 incorrect users. Univariate analysis showed that the gestational age of correct users was significantly higher than that of incorrect users (26.4 versus 22.4 weeks, *P* < 0.001, Table 2). There was a higher frequency of drivers who had received information about correct seatbelt wearing among correct seatbelt users than incorrect users (37.4% versus 26.3%, *P* = 0.003, Table 2).

**Table 2:** Backgrounds of correct and incorrect seatbelt users.

		Correct (n = 591)	Incorrect (n = 89)	P value
		Mean ± SD or n (%)	Mean ± SD or n (%)	
Age		31.5 ± 4.8	31.6 ± 5.3	0.248
BMI		20.6 ± 2.7	20.9 ± 2.4	0.752
Driving history (year)		11.1 ± 5.7	11.9 ± 6.1	0.155
Gestational age (weak)		26.4 ± 8.0	22.4 ± 8.3	< 0.001
Para	Primipara	319 (54.0%)	50 (56.2%)	0.733
	Multipara	272 (46.0%)	39 (43.8%)	
Understanding law regarding seat belt use	Correct	409 (69.3%)	58 (65.9%)	0.538
	Incorrect	181 (30.7%)	30 (34.1%)	
Type of vehicle	Sedan	158 (26.7%)	24 (27.0%)	0.729
	Small sedan (Kei-Car)	299 (50.6%)	48 (53.9%)	
	Support utility vehicle (SUV)	30 (5.1%)	2 (2.2%)	
	One-box car	96 (16.2%)	13 (14.6%)	
	Other	8 (1.4%)	2 (2.2%)	
Health guidance during pregnancy	Yes	43 (7.3%)	4 (4.5%)	0.500
	No	543 (92.3%)	85 (95.5%)	
Receiving information about correct seat belt use during pregnancy	Yes	221 (37.4%)	19 (21.3%)	0.003
	No	370 (62.6%)	70 (78.7%)	

To clarify the major factors that influenced correct seatbelt use, we undertook multivariate logistic regression analysis; we employed correct seatbelt use as a dependent variable and age, gestational age, BMI, primipara or multipara status, driving history, type of vehicle, understanding of the law, and receiving information about correct seatbelt use as independent variables. The results revealed that gestational age (odds ratio [OR], 1.06; confidence interval [95% CI], 1.03–1.09) and receiving information about correct seatbelt use during pregnancy (OR, 2.25; 95% CI, 1.31–3.87) were major independent factors for correct seatbelt use (Table 3).

**Table 3:** Multivariate analysis results for correct seatbelt use.

	Odds ratio	95% confidence interval	P
Gestational age	1.063	1.034–10.93	< 0.01
Receiving information about correct seatbelt use during pregnancy	2.252	1.312–3.867	0.003

## Discussion

We found that although most of the pregnant women drivers (97.6%) always wore a seatbelt, 12.7% of them did so incorrectly. The high rate of seatbelt use among our subjects is in accordance with the results of previous studies: over 90%^14,22,23^. One survey in 2001 suggested that the shoulder or lap belt was incorrectly used with 27.5% of pregnant women^24^. Our results are similar to those of that study.

Regarding the type of incorrect use, we found that the most common method was placing the shoulder belt across the protruded abdomen. With that usage, external forces are applied directly onto the abdomen through the tension in the belt. Another method that emerged in our survey involved placing the shoulder belt under the armpit and putting the lap belt over the thighs; that approach does not sufficiently restrict the driver, allowing easy forward movement and contact with the steering wheel.

A number of studies have reported incorrect seatbelt use by vehicle passengers. Among pregnant women drivers, it has been found that 13% placed the shoulder belt behind the shoulder, which is similar to our results^22^. It has been found that inappropriate seatbelt use can lead to intrauterine fetal death or milk-duct injury^25,26^. Further, even if passengers basically use seatbelts correctly, there are sometimes errors. A recent study of seatbelt position among rear-seat passengers found that the lap belt was placed above the correct position (on the anterior superior iliac spine) with 40% of males^27^. In such cases, the lap belt does not fit the ilium and compresses the abdomen; thus, such passengers may sustain serious abdominal organ injuries. Even when the seatbelt is used correctly, among short pregnant women sitting in the rear seat, there is occasional contact between the seatbelt and neck^28^. In that situation, adverse outcomes with neck compression by the shoulder belt have been simulated in frontal collision sled tests^29^. Therefore, it is necessary for health-care professionals to advise pregnant women about proper use of seatbelts.

It has been suggested that negative fetal outcomes may result from restrained pregnant women passengers involved in minor vehicle collisions. One report found that among fetal losses associated with trauma, 60%–70% of pregnant women suffered minor injuries^30^. One retrospective cohort study determined that even when pregnant women wore a seatbelt, preterm births occurred in 122 of 100,000 pregnancy days^31^. The same report observed 5.2 stillbirths, 7.0 placental abruptions, and 22.3 premature ruptures of the membranes^31^. From our results, we estimate that improper seatbelt use could contribute to negative fetal outcomes even in minor vehicle collisions.

One report based on real-world vehicle collisions involving pregnant women found that for a given crash severity, an 84% reduction in the risk of adverse fetal outcomes could be achieved by properly wearing a seatbelt^9^. Thus, appropriate seatbelt use should be a major prenatal counselling issue. The lap belt should be placed on the anterior superior iliac spine; the shoulder belt should be positioned to the side of the uterus, between the breasts, and over the mid-portion of the clavicle. However, in the present study, we found that only 35.6% of pregnant women drivers had received information about correct seatbelt use. Surprisingly, the great majority of our participants (93.2%) had not received any information about correct seatbelt use from health-care professionals. Therefore, health-care professionals in Japan should provide proper counselling about correct seatbelt use. Our multivariate analysis showed that receiving information about correct seatbelt use and gestational age were significant independent factors for appropriate use. Our results underline the importance of counselling about correct seatbelt use early in pregnancy.

From the high seatbelt use rate (97.6%) observed in the present study, we conclude that most pregnant women understand the importance of such use. However, in the present study, 31.8% of drivers did not properly understand the law concerning seatbelt use; notably, 23.6% of drivers had the mistaken belief that seatbelt use was not legally required for rear-seat passengers. This finding is in accordance with those of one report, which determined that rear-seat passengers were significantly less likely always to wear a seatbelt than front-seat passengers^20^. Thus, when counselling about seatbelt use, health-care professionals should provide information about the law regarding seatbelt use. Some pregnant women occupying a rear seat reportedly dislike wearing a seatbelt owing to discomfort^28,29^. Accordingly, future studies about pregnant women sitting in a rear seat should be performed for comparison with the results of the present study.

This study has several limitations. First, the survey response rate was 77.4%, which may limit generalizability about pregnant women in Shiga Prefecture. However, during the study period, the estimated number of pregnant women in south-west Shiga Prefecture was 4,580; thus, approximately one in six pregnant women in that area responded to the survey. Accordingly, for a population-based survey of pregnant women, we believe we obtained sufficiently reliable results. Second, we did not directly ask about the importance of seatbelt use. To ascertain participants’ understanding more accurately in this regard, it would be necessary to ask more detailed questions, such as with respect to the benefits and harms of a particular type of behaviour. However, we focused on incorrect seatbelt use and related factors; thus, we do not believe that this issue could have influenced our results. Third, when using a seatbelt, some pregnant women feel discomfort. Some reports have suggested that discomfort when wearing a seatbelt was a reason for not using one^32,33^. Most of the pregnant women in the present study wore seatbelts; however, we did not examine the relationship between discomfort and incorrect seatbelt use. Future research should focus on perceptions among pregnant women when driving a vehicle.

We believe that the findings of this study will be beneficial for health-care professionals in advising pregnant patients always to wear a three-point seatbelt correctly when travelling in vehicles.
